# Incremental Value of ePLAR—The Echocardiographic Pulmonary to Left Atrial Ratio in the Assessment of Sub-Massive Pulmonary Emboli

**DOI:** 10.3390/jcm9010247

**Published:** 2020-01-17

**Authors:** Isabel G. Scalia, William M. Scalia, Jonathon Hunter, Andrea Z. Riha, David Wong, Yael Celermajer, David G. Platts, Benjamin T. Fitzgerald, Gregory M. Scalia

**Affiliations:** 1 Royal Brisbane and Women’s Hospital, Herston, QLD 4029, Australia; igscalia@gmail.com (I.G.S.); yaelgcelermajer@gmail.com (Y.C.); 2 Department of Medicine, University of Queensland, Brisbane, QLD 4032, Australia; dgplatts@hotmail.com; 3 The Prince Charles Hospital, Brisbane, QLD 4032, Australia; will.scalia2@gmail.com; 4 Redcliffe District Hospital, Redcliffe, QLD 4032, Australia; jonhunter749@icloud.com; 5 The Wesley Hospital, Brisbane, QLD 4066, Australia; azriha@hotmail.com (A.Z.R.); dcwong@iprimus.com.au (D.W.); bmcd124@yahoo.com.au (B.T.F.); 6 Genesis Care, Auchenflower, QLD 4066, Australia

**Keywords:** pulmonary embolus, echocardiography, ePLAR

## Abstract

Background: Acute pulmonary embolism (PE) is characterized hemodynamically by abrupt obstruction in trans-pulmonary blood flow. The echocardiographic Pulmonary to Left Atrial ratio (ePLAR, tricuspid regurgitation V_max_/mitral E/e’) has been validated as a non-invasive surrogate for trans-pulmonary gradient (TPG) that accurately differentiates *pre-capillary* from *post-capillary* chronic pulmonary hypertension. This study assessed ePLAR as an incremental echocardiographic assessment tool compared with traditional measures of right ventricular pressure and function. Methods: In total, 110 (57.4 ± 17.6 years) patients with confirmed sub-massive pulmonary emboli with contemporaneous echocardiograms (0.3 ± 0.9 days) were compared with 110 age-matched controls (AMC). Results: Tricuspid velocities were higher than AMC (2.6 ± 0.6 m/s vs. 2.4 ± 0.3 m/s, *p* < 0.05), although still consistent with “normal” right ventricular systolic pressures (34.2 ± 13.5 mmHg vs. 25 ± 5.3 mmHg, *p* < 0.05) with lower mitral E/e’ values (8.2 ± 3.8 vs. 10.8 ± 5.1, *p* < 0.05). ePLAR values were higher than AMC (0.36 ± 0.14 m/s vs. 0.26 ± 0.10, *p* < 0.05) suggesting significantly elevated TPG. Detection of abnormal echocardiographic findings increased from 29% (TRV_max_ ≥ 2.9 m/s) and 32% (reduced tricuspid annular plane systolic excursion) to 70% with ePLAR ≥ 0.3 m/s. Conclusions: Raised ePLAR values in acute sub-massive pulmonary embolism suggest elevated trans-pulmonary gradients even in the absence of acutely increased pulmonary artery pressures. ePLAR dramatically increases the sensitivity of echocardiography for detection of hemodynamic perturbations in sub-massive pulmonary embolism patients, which may offer clinical utility in diagnosis and management.

## 1. Background

Clinically, presentations of acute pulmonary embolism typically include acute progressive shortness of breath with associated pleuritic chest pain, with or without cough/hemoptysis. Physiologic derangements manifest as tachycardia, syncope/presyncope, and in severe cases may result in systemic hypotension, hemodynamic instability, or shock. Physiologically, this phenomenon occurs due to a sudden impairment of blood flow from the right heart through the lungs to the left heart with a consequent reduction in systemic blood flow. Echocardiographically, the abrupt increase in trans-pulmonary gradient leads to acute right ventricular dysfunction (often with the classic distribution of McConnell’s sign), with or without increased right ventricular systolic pressure. Obstructed trans-pulmonary flow leads to a decrease in left atrial filling pressure, with consequently reduced left ventricular filling that may result systemic hypo-perfusion and/or cardiogenic shock.

Acute pulmonary embolism (PE) presentations carry a mortality rate as a high as 25% in the first 30 days [1]. As such, prompt and thorough evaluation and management is crucial in minimizing the potential morbidity and mortality associated [2]. The “gold standard” diagnosis of acute pulmonary embolism is via computed tomography pulmonary angiogram (CTPA), to anatomically assess clot burden and distribution [3]. Ventilation perfusion scintigraphy (V/Q scan) has comparative utility, via assessment of the burden of mismatched perfusion defects, as a diagnostic and prognostic measure [4]. The hemodynamic functional effect of PE however is typically assessed by echocardiography [5]. Specifically, patients are assessed for evidence of right ventricular (RV) dysfunction/enlargement and/or significant pulmonary hypertension, manifest as elevated right ventricular systolic pressures [6]. Based on European Society of Cardiology Guidelines 2014 [7], immediate thrombolysis is both justified and recommended in the setting of significant RV dysfunction and associated cardiogenic shock or hemodynamic instability. This includes instances where CTPA may not be feasible prior to echocardiographic evaluation. 

In this setting, significant right ventricular dysfunction may be evidenced by reduced right ventricular function, e.g., McConnell’s sign (RV free wall akinesia with apical wall hypercontractility) [8], reduced TAPSE (tricuspid annular plane systolic excursion) [9], reduced tricuspid annular Doppler Tissue Imaging S’-velocity (RV S’) [10], and, more recently, reduced RV free wall longitudinal strain [11,12]. All of these are functional assessments, which suggest right ventricular dysfunction in the setting of acutely increased afterload. Importantly, however, the absence of RV dysfunction on initial echocardiography does not definitively exclude significant embolic burden in hemodynamically, normotensive stable patients [7]. Furthermore, these measurements of RV dysfunction may be confounded by RV pathology (for example with RV wall infarct, or pre-existing pulmonary hypertension) [13]. Conversely, in hemodynamically unstable patients with suspected high risk PE, the absence of echocardiographic evidence of RV dysfunction or overload can effectively exclude PE [5].

**Figure 1:**
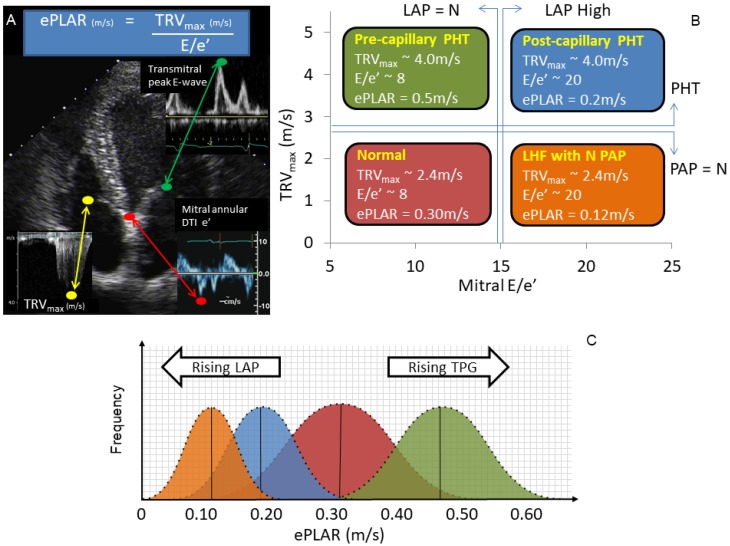
ePLAR (echocardiographic Pulmonary to Left Atrial ratio = tricuspid regurgitation V_max_/mitral E/e’), explanation and example data. (**A**) The ePLAR comprises three simple measurements: peak tricuspid regurgitation continuous wave velocity (TRV_max_) (m/s) divided by the trans-mitral peak pulsed wave Doppler E wave (cm/s) Peak Doppler Tissue Imaging mitral septal annular e’ wave (E/e’) (cm/s). (**B**) The four nominal patient subsets clinically encountered are demonstrated with predicted bell curves displayed. (**C**) Normal cases (**red**) will have normal pulmonary artery pressure (PAP) and left atrial pressure (LAP), normal TRV_max_ (e.g., 2.4 m/s), normal E/e’ (e.g., 8), and a predicted ePLAR of approximately 0.30 m/s. Patients with left heart failure (LHF) but with normal pulmonary arterial pressures (PAP_mean_ < 25 mmHg) will have normal TRV_max_ (e.g., 2.4 m/s) with a high E/e’ (e.g., 20), yielding an ePLAR of approximately 0.12 m/s (**orange**). Patients with *post-capillary* pulmonary hypertension secondary to LHF will have a high TRV_max_ (e.g., 4.0 m/s) and a high E/e’ (e.g., 20), yielding an ePLAR of approximately 0.2 m/s (**blue**). Patients with *pre-capillary* pulmonary hypertension will have a high TRV_max_ (e.g., 4.0 m/s) with a normal E/e’ (e.g., 8), yielding the highest of ePLAR values-approximately 0.50 m/s in this example (**green**). (**C**) ePLAR will be higher than normal in patients with *pre-capillary* physiology (rising TPG) and lower than normal in patients with *post-capillary* physiology (rising LAP). Figure reproduced with permission [14].

The pathophysiologic underpinning of the hemodynamic perturbation of acute PE is *pre-capillary* obstruction to trans-pulmonary flow. The novel parameter ePLAR (the echocardiographic Pulmonary to Left Atrial Ratio) has been validated as a non-invasive surrogate to trans-pulmonary gradient [14]. ePLAR, which assesses the relationship between right ventricular systolic pressure and left atrial pressure via the formula,

ePLAR _(m/s)_ = TRV_max (m/s)_/mitral E/e’ (1)

has been shown to correlate well with invasively-derived trans-pulmonary gradient (TPG). ePLAR non-invasively differentiates between *pre-capillary* and *post-capillary* pulmonary hypertension in patients being investigated for consideration of specific pulmonary vasodilator therapies. Higher ePLAR values suggest higher trans-pulmonary gradients and *pre-capillary* pulmonary hypertension. Lower ePLAR values suggest higher left atrial pressures with minimal increase in trans-pulmonary gradient and *post-capillary* pulmonary hypertension (see Figure 1 [14]). It has also been shown that with age, as left heart filling pressures naturally rise, ePLAR declines linearly. Thus, consideration of normal vs. abnormal values in a given patient must take into consideration age.

It is hypothesized that ePLAR (as a measure of trans-pulmonary gradient) may have a higher diagnostic yield than other previously utilized echocardiographic markers of elevated pulmonary pressures (as assessed by TRV_max_/RVSP) and RV dysfunction (reduced TAPSE or RV S’) in detecting hemodynamic perturbations in patients with acute sub-massive pulmonary embolism. Abnormally high ePLAR levels would likely be comprised of a small (if any) increase in TRV_max_ with significantly reduced mitral E/e’ (consistent with reduced left atrial filling pressures). Increased ePLAR in these patients will suggest increased TPG even in the absence of actual elevated right heart pressures or right ventricular dysfunction. 

## 2. Methods

### 2.1. Patient Inclusion

Consecutive patients referred to three Major Australian Hospital echocardiography laboratories with pulmonary emboli confirmed by CT pulmonary angiography (CTPA) or ventilation/perfusion (V/Q) scintigraphy were studied. Patients with massive embolic load associated with shock requiring resuscitation were not included in this study. All remaining patients were shown to have sub-massive embolic burden (bilateral or saddle). All patients were studied at the time of diagnosis (< ±72 h) with comprehensive echocardiography. Human Research and Ethics Committee approval for review of data was obtained from each institution. A further cohort of age-matched, paired “normal subjects” was generated with permission from the echocardiography database of the referring institutions. These subjects were defined as normal via the criteria: normal left ventricular size and function (systolic and diastolic), normal right ventricular size and function, normal intracardiac valves, and no clinical history of pulmonary embolism, lung disease, or pulmonary arterial hypertension.

### 2.2. Echocardiographic Methods

All patients referred for transthoracic echocardiography with the diagnosis of suspected pulmonary embolism were studied with comprehensive 2D, Doppler and strain analysis (where available). Left heart function, size, and valvular function was assessed as per standard protocols. Left heart diastolic filling was assessed using pulsed wave Doppler at the mitral tips according to the ASE guidelines [15] Mitral annular Doppler Tissue Imaging (DTI) velocities were assessed in the annulus (septal and lateral). Right heart function was assessed using tricuspid annular plane systolic excursion (TAPSE) measured from color DTI M-mode. Tricuspid annular systolic DTI peak velocity (RV S’) was obtained by placing a cursor slightly distal to the lateral annulus in the four-chamber view. All measurements were averaged over three beats in sinus rhythm and five beats in atrial fibrillation.

### 2.3. Statistical Methods

**Figure 2:**
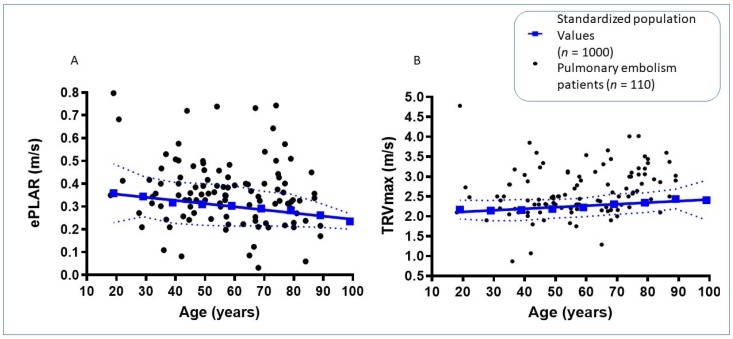
(**A**) Age stratified echocardiographic ePLAR values (calculated as TRV_max_/mitral E/e’) for CTPA or V/Q scan confirmed acute pulmonary embolism patients (*n* = 110) vs. ePLAR of previously documented “standardized population” [14]. (**B**) Age stratified echocardiographically obtained TRV_max_ values for patients with confirmed PE vs. “standardized normal population”.

Linear measurements were assessed using normalized ratios and student t-tests. Parametric variables were evaluated using the Chi squared student test. Significance was defined by *p* < 0.05. Graphical patient data were compared with a previously generated “standardized population” of 1000 subjects as reference for ePLAR and TRV_max_ values, stratified by decade (see Figure 2) [14]. Comparisons of the patients with their age-matched controls was used to generate sensitivity and specificity for each echocardiographic parameter in predicting the presence of pulmonary embolism. Receiver operator curves (ROC) curves were generated using GraphPad Prism 8, via the Hanley method, to compare the predictive power of tested parameters for pulmonary embolism.

## 3. Results

There were 223 patients referred for acute echocardiography with the diagnosis of possible/probable/proven sub-massive pulmonary embolism. Of these, 88 patients were subsequently shown to either not have sub-massive pulmonary emboli (32 patients with small/lobar pulmonary emboli and 56 with negative CTPA/VQ scans for PE) or had a delay of >72 h from CTPA/VQ to echocardiography. A further 25 patients were excluded with incomplete datasets. The remaining 110 patients (64 male, aged 57.4 ± 17.6 years) were shown to have bilateral pulmonary emboli (*n* = 97) or saddle emboli (*n* = 13) by CTPA/VQ scan. Echocardiography was performed at 0.3 ± 0.9 days from CTPA/VQ (range −3 to +3 days). Demographics of the patients are shown in Table 1, with statistical comparison to the aged matched control cohort.

Based on recent guideline definitions of pulmonary hypertension [12], a TRV_max_ > 2.9 m/s was defined as indicative of elevated right ventricular systolic pressure. Based on the previous validation of ePLAR [14], values > 0.28 m/s were considered to be indicative of elevated TPG. Normal values for TAPSE were considered excursions distances ≥17 mm. Normal values for tricuspid annular Doppler Tissue Imaging systolic velocity (RV S’) were considered ≥9.5 cm/s [16].

**Figure 3:**
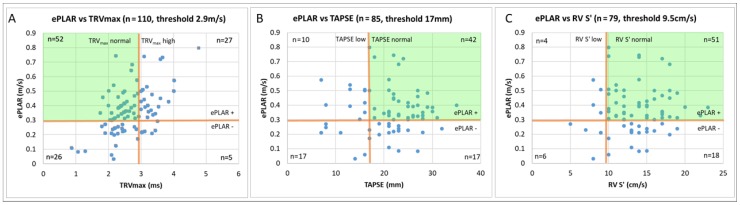
Graphical display of the positive and negative diagnostic yield of ePLAR (ePLAR+ defined as ePLAR > 0.3 m/s, ePLAR-defined as ePLAR ≤ 0.3 m/s) versus TRV_max_ ((**A**) TRV_max_ normal defined as TRV_max_ ≤ 2.9 m/s, high TRV_max_ > 2.9 m/s), TAPSE ((**B**) TAPSE normal defined as TAPSE ≥ 17 mm, low TAPSE < 17 mm), and RV S’ ((**C**) RV S’ normal defined as RV S’ ≥ 9.5 cm/s, low RV S’ < 9.5 cm/s). Incremental diagnostic yield of ePLAR (green shaded quadrants) represented as patients with true positive ePLAR and false negative relative comparison parameter.

Using these cut-off values, patients were separated analyzed in dichotomous assessments of ePLAR vs. TRV_max_ (Table 2 and Figure 3A), ePLAR vs. TAPSE (Table 3 and Figure 3B), and ePLAR vs. RV S’ (Table 4 and Figure 3C). For each respective analysis, four groups were defined: Group 1 (abnormal TRV_max_ or TAPSE or RV S’, elevated ePLAR ≥ 0.28 m/s), Group 2 (normal TRV_max_ or TAPSE or RVS’, elevated Eplar ≥ 0.28 m/s), Group 3 (abnormal TRV_max_ or TAPSE or RVS’, normal ePLAR < 0.28 m/s), and Group 4 (normal TRV_max_ or TAPSE or RVS’, normal ePLAR < 0.28 m/s). 

**Figure 4:**
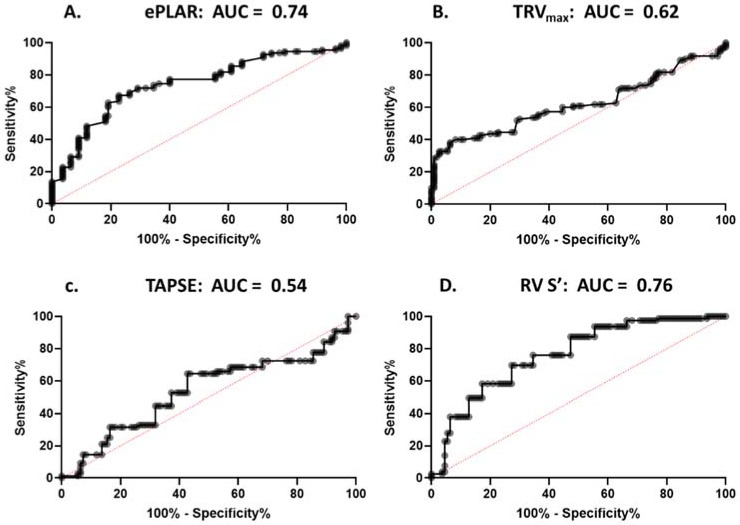
Receiver operator curves assessing predict power of each parameter (ePLAR, TRV_max_, TAPSE, and RV S’) in predicting pulmonary embolism, when compared to age-matched controls.

Using the standardized cut–off values listed above, sensitivity and specificity data were calculated for the prediction of pulmonary embolism, in comparison to the age matched control cohort. As shown in Table 5, ePLAR performed most strongly with sensitivity 72% (confidence interval (CI) 62–80%), specificity 66% (CI 57–75%), positive predictive value 68% (CI 62–74%), and negative predictive value 70% (CI 63–75%). TRV_max_ demonstrated sensitivity 29% (CI 21–39%), specificity 98% (CI 94–100%), positive predictive value 94% (CI 80–98%), and negative predictive value 58% (CI 57–70%). Right ventricular function as assessed by TAPSE demonstrated sensitivity 22% (CI 14–33%), specificity 85% (CI 77–91%), positive predictive value 52% (CI 36–66%), and negative predictive value 61% (CI 58–65%). Right ventricular function as assessed by RV S’ demonstrated sensitivity 13% (CI 6–22%), specificity 85% (CI 76–91%), positive predictive value 37% (CI 22–55%), and negative predictive value 57% (CI 55–60%). Comparison of the predictive power of each parameter was assessed using ROC curves (see Figure 4). ePLAR performed strongly (AUC 0.74, *p* < 0.05) compared with TRV_max_ (AUC 0.62, *p* < 0.05), TAPSE (AUC 0.54, *p* = 0.33), and RV S’ (AUC 0.76, *p* < 0.05).

## 4. Discussion

Sub-massive acute pulmonary embolism is associated with significant morbidity and mortality, in both the acute and chronic setting. It has been well documented that early treatment with anticoagulation, and in severe cases thrombolysis, is critical in the care of these patients [2]. Anatomical assessment of clot location and burden is well achieved with CTPA and nuclear medicine V/Q scan in most cases. Echocardiography is unlikely to ever replace these tests for definitive diagnosis of PE [5,17].

However, echocardiography has two major roles in this disease spectrum [7]. Firstly, in some situations, these anatomic tests are logistically not feasible in the emergent setting, and echocardiography is used to assess for “supportive” or “surrogate” evidence to confirm or refute the clinical suspicion of PE [18]. Indeed, while clearly not the optimal test for the definitive diagnosis of PE, echocardiography is frequently requested (often before CTPA or V/Q scans can logistically be obtained) to guide emergent therapy. Clinicians have long recognized the constellation of visual cues (McConnell’s sign with preserved apical RV function [8] and reduced free wall function), in the setting of minimally elevated TRV_max_/RVSP values, and underfilled left-sided chambers. Quantitative measures of RV function help reinforce these subjective impressions of the constellation of findings of significant PE [10]. 

In this study, patients with proven acute PE had mean TAPSE values of 21.1 mm and RV S’ of 13.5 cm/s, with 59/110 and 69/110 false negative results, respectively. As a binary diagnostic tool for RV dysfunction, TRV_max_ performed poorly, with only 32 out of 110 patients having appropriately elevated TRV_max_ ≥ 2.9 m/s. Clinically, all three parameters had very low sensitivity for the detection of acute PE (TRV_max_ 29%, TAPSE 22%, RV S’ 13%), though high specificity. ePLAR on the other hand was found to have much higher sensitivity (72%) with modest specificity (66%), 31/110 results being falsely normal. Although RV S’ was found to have a greater ROC AUC than ePLAR, ePLAR was found to have the highest negative predictive value of all parameters, and much higher positive predictive value than RV S’. Clinically, these data suggest that ePLAR, as a marker of impaired trans-pulmonary flow, is much more likely to register as abnormal than measures of right heart pressure (RVSP/TRV_max_), or right heart function (TAPSE and RV S’), with a negative predictive value superior to these other parameters.

Secondly, and more logically, echocardiography in the setting of suspected or proven acute PE plays a major role in risk stratification and the assessment of hemodynamic distress of the right-sided circulation. Current Guidelines (both European and American) for thrombolysis or catheter-based intervention for PE recommend echocardiographic assessment of RV dysfunction [7,19], and subsequent treatment in the setting of RV dysfunction + hemodynamic instability, with or without CTPA or V/Q scan confirmation of PE [7]. Prognostic advantage has been demonstrated when these criteria lead to lysis/intervention [20]. However, in this study, only 32% had reduced TAPSE while 70% had abnormal ePLAR. It is hypothesized that ePLAR is a subtler indicator of hemodynamic disturbance. Future studies may well show that using this marker of impaired transpulmonary flow may better predict response to therapy. 

Finally, this study offers mechanistic insights into the hemodynamics of acute PE, and, specifically, the physiologic underpinning of systemic hypotension and shock [21]. In this cohort, the mitral E/e’ (as a marker of left atrial pressure) was substantially lower than age-matched controls (8.2 ± 3.8 vs. 10.8 ± 5.1, *p* = 0.013). Even though, as a group, these pulmonary embolism patients were not pulmonary hypertensive (TRV_max_ 2.61 ± 0.61 m/s, RVSP 34.18 ± 13.49 mmHg), ePLAR was markedly elevated, suggesting for the first time non-invasively, elevated TPG levels. Interestingly, for each of the three echocardiographic parameters assessed dichotomously with ePLAR (Table 2, Table 3 and Table 4), patients in Group 2 (ePLAR true positive, TRV_max_/TAPSE/RV S’ false negative) were significant younger than patients in Group 1 or 3 (TRV_max_/TAPSE/RV S’ true positive). This may suggest an element of age related physiologic compensation or pulmonary vasculature reserve in the setting of an acute obstruction to trans-pulmonary flow [22]. Consequently, it is hypothesized that ePLAR may offer mechanistic understanding into normalization of trans-pulmonary flow in acute PE following therapy [23]. Follow up echocardiographic analysis may also provide insight into the long-term physiologic impacts of acute PE, in particular in the setting of pre-existing cardiac systolic or diastolic dysfunction.

## 5. Limitations

It should be noted that this a retrospective study, over several years, of a very large cohort of patients presenting with suspected or confirmed pulmonary embolus. Clinical practice varies from case to case, and across time and institutions. The subset of patients with complete datasets, adequate for calculation of all required parameters for this study, is reflected in the size of the final cohort. The age-matched control group was selected randomly from a very large database of normal individuals. The groups are reasonably well matched, but this is never a perfect process. The delay between echo and anatomic imaging study was set at a maximum of ±3 days. It could be well argued that an echocardiogram performed three days after diagnosis and onset of anticoagulation for PE may well have some dilution of the hemodynamic effects of the condition. 

## 6. Conclusions

The majority of patients with sub-massive pulmonary embolism, in the absence of shock, demonstrated normal estimated right ventricular systolic pressure and normal right ventricular function. Despite this, these patients exhibited a significant increase in the echocardiographic Pulmonary to Left Atrial Ratio (ePLAR), suggesting increased trans-pulmonary gradient with *pre-capillary* obstruction to flow. This new parameter may well offer a more sensitive echocardiographic sign of abnormal hemodynamic disturbance in these patients. Future studies should focus on the value of ePLAR in acute patient triage, guidance of therapy, and prediction of prognosis in the setting of acute pulmonary embolism.

## Figures and Tables

**Table 1:** Demographics of pulmonary embolism patients compared with age-matched control cohort.

	PE Patients	Age-Matched Controls	Significance (*p* < 0.05)
*n*	110	110	-
Age (years)	57.4 ± 17.6	58.1 ± 17.8	0.76
Male (%)	58	60	-
BSA (m^2^)	2.1 ± 0.3	1.98 ± 0.27	0.01
Systolic blood pressure (mmHg)	124.9 ± 15.7	121.5 ± 20.6	0.42
Diastolic blood pressure (mmHg)	74.3 ± 8.5	74.2 ± 7.4	0.93
TRV_max_ (m/s)	2.61 ± 0.61	2.36 ± 0.28	0.0001
ePLAR (m/s)	0.36 ± 0.14	0.26 ± 0.10	<0.0001
RVSP (mmHg)	34.18 ± 13.49	25 ± 5.3	<0.0001
TAPSE (mm)	21.08 ± 6.31	20.60 ± 5.91	0.60
RV S’ (cm/s)	13.49 ± 3.58	12.6 ± 3.3	<0.0001
Mitral E/e’	8.2 ± 3.8	10.8 ± 5.1	0.01

PE, pulmonary embolus; BSA, body surface area; TRV, Tricuspid regurgitation maximum continuous-wave Doppler velocity; ePLAR, echocardiographic Pulmonary to Left Atrial Ratio (ePLAR = tricuspid regurgitation V_max_/mitral E/e’; RVSP, right ventricular systolic pressure; TAPSE, tricuspid annular plane systolic excursion; RV S’, right ventricular peak Doppler Tissue Imaging systolic velocity.

**Table 2:** Demographic and echocardiographic evaluation by Group ePLAR vs. TRV_max_.

	Group 1	Group 2	Group 3	Group 4
*N*	27	48	5	30
Age (years)	62.95 ± 17.54	54.89 ± 16.03	77.77 ± 9.47	53.02 ± 18.31
Male (%)	63	60	20	57
BSA (m^2^)	2.14 ± 0.34	2.10 ± 0.27	1.97 ± 0.3	2.08 ± 0.42
Baseline HR (bpm)	76.8 ± 20.7	75.2 ± 11.84	66.0 ± 9.2	67.74 ± 18.31
Time to echo (days)	0.22 ± 1.09	0.23 ± 0.66	0.60 ± 0.89	0.53 ± 1.04
TRV_max_ (m/s)	3.38 ± 0.42	2.42 ± 0.26	3.19 ± 0.19	2.11 ± 0.44
ePLAR (m/s)	0.48 ± 0.08	0.42 ± 0.13	0.20 ± 0.08	0.20 ± 0.07
TAPSE (mm)	18.17 ± 5.46	24.23 ± 6.57	18.25 ± 3.87	19.79 ± 6.43
S’(cm/s)	12.68 ± 3.50	14.44 ± 3.95	12.00 ± 3.87	13.17 ± 2.85

Group 1 (Abnormal TRV_max_, elevated ePLAR ≥ 0.28 m/s), Group 2 (normal TRV_max_, elevated ePLAR ≥ 0.28 m/s), Group 3 (Abnormal TRV_max_, normal ePLAR < 0.28 m/s), and Group 4 (normal TRV_max_, normal ePLAR < 0.28 m/s).

**Table 3:** Demographic and echocardiographic evaluation by Group ePLAR vs. TAPSE.

	Group 1	Group 2	Group 3	Group 4
*N*	5	47	6	18
Age (years)	72.64 ± 7.81	54.59 ± 18.28	59.50 ± 19.67	60.82 ± 19.41
Male (%)	20	55	67	44
BSA (m^2^)	2.08 ± 0.3	2.09 ± 0.35	1.97 ± 0.31	1.98 ± 0.34
Baseline HR (bpm)	68.0 ± 9.1	73.1 ± 10.26	66.0 ± 8.0	62.8 ± 8.18
Time to echo (days)	0.20 ± 2.17	0.32 ± 0.81	0.00 ± 0.3	1.06 ± 1.21
TRV_max_ (m/s)	3.20 ± 0.69	2.72 ± 0.59	2.39 ± 0.48	2.22 ± 0.72
ePLAR (m/s)	0.47 ± 0.14	0.40 ± 0.10	0.22 ± 0.01	0.21 ± 0.08
TAPSE (mm)	10.80 ± 3.03	23.30 ± 4.66	9.17 ± 2.79	22.11 ± 4.07
S’ (cm/s)	9.80 ± 1.79	14.48 ± 3.43	8.00 ± 1.73	13.67 ± 2.91

Group 1 (Abnormal TAPSE, elevated ePLAR ≥ 0.28 m/s), Group 2 (normal TAPSE, elevated ePLAR ≥ 0.28 m/s), Group 3 (Abnormal TAPSE, normal ePLAR < 0.28 m/s), and Group 4 (normal TAPSE, normal ePLAR < 0.28 m/s).

**Table 4:** Demographic and echocardiographic evaluation by Group ePLAR vs. RV S’.

	Group 1	Group 2	Group 3	Group 4
*N*	4	51	6	18
Age (years)	62.75 ± 17.86	54.76 ± 18.26	64.17 ± 20.83	61.32 ± 20.03
Male (%)	25	53	67	44
BSA (m^2^)	2.14 ± 0.32	2.07 ± 0.33	1.97 ± 0.3	1.98 ± 0.34
Baseline HR (bpm)	76.8 ± 20.7	75.2 ± 14.93	66.0 ± 9.1	71.3 ± 8.18
Time to echo (days)	−0.25 ± 1.89	0.33 ± 0.86	0.33 ± 0.82	0.94 ± 1.21
TRV_max_ (m/s)	3.26 ± 0.54	2.72 ± 0.59	2.60 ± 0.63	2.19 ± 0.68
ePLAR (m/s)	0.46 ± 0.10	0.42 ± 0.13	0.19 ± 0.08	0.20 ± 0.07
TAPSE (mm)	13.0 ± 6.68	22.8 ± 5.17	10.7 ± 4.13	22.4 ± 3.99
S’ (cm/s)	8.5 ± 0.58	14.3 ± 3.32	7.8 ± 1.60	14.1 ± 2.40

Group 1 (Abnormal RV S’, elevated ePLAR ≥ 0.28 m/s), Group 2 (normal RV S’, elevated ePLAR ≥ 0.28 m/s), Group 3 (Abnormal RV S’, normal ePLAR < 0.28 m/s), and Group 4 (normal RV S’, normal ePLAR < 0.28 m/s).

**Table 5:** Sensitivity and specificity of each echocardiographic parameter in the detection of acute pulmonary embolism compared to age-matched controls.

Percentage (95% CI)	Sensitivity	Specificity	Positive Predictive Value	Negative Predictive Value
ePLAR	72% (62–80%)	66% (57–75%)	68% (62–74%)	70% (63–75%)
TRV_max_	29% (21–39%)	98% (94–100%)	94% (80–98%)	58% (57–70%)
TAPSE	22% (14–33%)	85% (77–91%)	52% (36–66%)	61% (58–65%)
RV S’	13% (6–22%)	85% (76–91%)	37% (22–55%)	57% (55–60%)

## Abbreviations

PE	Pulmonary Embolus
RV	Right ventricle
TAPSE	Tricuspid annular plane systolic excursion
RV S’	Right ventricular DTI systolic velocity (measured at the tricuspid annulus)
PAP	Pulmonary artery pressure
LAP	Left atrial pressure
TPG	Trans-pulmonary gradient
RVSP	Right ventricular systolic pressure
TRV_max_	Tricuspid regurgitation maximum continuous-wave Doppler velocity
ePLAR	echocardiographic Pulmonary to Left Atrial Ratio
CTPA	Computed tomography pulmonary angiogram
DTI	Doppler Tissue Imaging
Mitral E/e’	Pulsed wave Doppler peak trans-mitral e-wave velocity/DTI mitral annular e’-wave velocity
